# Transforming Growth Factor-*β* Protects against Inflammation-Related Atherosclerosis in South African CKD Patients

**DOI:** 10.1155/2018/8702372

**Published:** 2018-06-06

**Authors:** Muzamil Olamide Hassan, Raquel Duarte, Therese Dix-Peek, Caroline Dickens, Sagren Naidoo, Ahmed Vachiat, Sacha Grinter, Pravin Manga, Saraladevi Naicker

**Affiliations:** ^1^Division of Nephrology, Department of Internal Medicine, Faculty of Health Sciences, University of the Witwatersrand, South Africa; ^2^Internal Medicine Research Laboratory, Department of Internal Medicine, Faculty of Health Sciences, University of the Witwatersrand, South Africa; ^3^Division of Cardiology, Department of Internal Medicine, Faculty of Health Sciences, University of the Witwatersrand, South Africa; ^4^Department of Internal Medicine, Faculty of Health Sciences, University of the Witwatersrand, South Africa

## Abstract

**Background:**

Transforming growth factor-*β* (TGF-*β*) may inhibit the development of atherosclerosis. We evaluated serum levels of TGF-*β* isoforms concurrently with serum levels of endotoxin and various inflammatory markers. In addition, we determined if any association exists between polymorphisms in the* TGF-β1* gene and atherosclerosis in South African CKD patients.

**Methods:**

We studied 120 CKD patients and 40 healthy controls. Serum TGF-*β*1, TGF-*β*2, TGF-*β*3, endotoxin, and inflammatory markers were measured. Functional polymorphisms in the* TGF-β1* genes were genotyped using a polymerase chain reaction-sequence specific primer method and carotid intima media thickness (CIMT) was assessed by B-mode ultrasonography.

**Results:**

TGF-*β* isoforms levels were significantly lower in the patients with atherosclerosis compared to patients without atherosclerosis (p<0.001). Overall, TGF-*β* isoforms had inverse relationships with CIMT. TGF-*β*1 and TGF-*β*2 levels were significantly lower in patients with carotid plaque compared to those without carotid plaque [TGF-*β*1: 31.9 (17.2 – 42.2) versus 45.9 (35.4 – 58.1) ng/ml, p=0.016; and TGF-*β*2: 1.46 (1.30 – 1.57) versus 1.70 (1.50 – 1.87) ng/ml, p=0.013]. In multiple logistic regression, age, TGF-*β*2, and TGF-*β*3 were the only independent predictors of subclinical atherosclerosis in CKD patients [age: odds ratio (OR), 1.054; 95% confidence interval (CI): 1.003 – 1.109, p=0.039; TGF-*β*2: OR, 0.996; 95% CI: 0.994–0.999, p=0.018; TGF-*β*3: OR, 0.992; 95% CI: 0.985–0.999, p=0.029).* TGF-β1* genotypes did not influence serum levels of TGF-*β*1 and no association was found between the* TGF-β1* gene polymorphisms and atherosclerosis risk.

**Conclusion:**

TGF-*β* isoforms seem to offer protection against the development of atherosclerosis among South African CKD patients.

## 1. Introduction

Chronic kidney disease (CKD) patients are more likely to develop cardiovascular disease (CVD) than age-matched counterparts in the general population. As a consequence, the risk of death in CKD patients due to cardiovascular disease is much higher than the risk of requiring dialysis [1, 2].

Chronic inflammation is directly related to several complications of CKD, including accelerated atherosclerosis and left ventricular hypertrophy [2, 3]. Chronic low-grade inflammation is common in patients with coexisting CKD and CVD and plays a pivotal role in the development of atherosclerotic plaques by driving oxidative stress and stimulating production of inflammatory cytokines leading to activation of chemokines and adhesion molecules [3–5].

Endotoxin (lipopolysaccharide), a glycolipid that comprises most of the outer wall of gram-negative bacteria, is a potential source of inflammation in CKD patients [6, 7]. It is reported that circulating endotoxaemia constitutes a strong risk factor for atherosclerotic CVD [8–10]. This finding suggests that chronic exposure to endotoxins may be related to subclinical atherosclerosis and represents a reversible CVD risk factor in CKD patients.

Atherosclerosis is a complex disease process in which inflammation plays a central role in various pathogenetic mechanisms that contribute to the progressive structural changes that are characteristic of atherogenesis [11, 12]. Besides promoting atherosclerosis, inflammation also plays a significant role in the process of plaque rupture and arterial thrombosis, leading to vascular occlusion and infarction [13]. Thus, inflammation has been found to be a significant predictor of cardiovascular mortality in CKD patients [14].

Transforming growth factor-*β* (TGF-*β*), a multifunctional inflammatory cytokine, is produced by many inflammatory cells including leucocytes, macrophages, smooth muscle cells, and platelets [15–18]. There are three isoforms of TGF-*β*: TGF-*β*1, TGF-*β*2, and TGF-*β*3. Transforming growth factor-*β*1, the most extensively studied of these three isoforms, exhibits anti-inflammatory and antiproliferative properties by inhibiting the synthesis of tumour necrosis factor-*α* (TNF-*α*) or by downregulating the proinflammatory effects of IL-1*β* and interferon-*γ* [19, 20]. In turn, this leads to reduction of inflammatory cytokine-induced vascular cell adhesion molecule-1 (VCAM-1), chemotaxis, leucocyte adhesion to vascular endothelial lining, and decreased macrophage activity [19, 21]. Previous studies have suggested that low serum levels of TGF-*β*1 are a risk factor for atherosclerosis in non-CKD [22, 23] and CKD patients [10]. However, there is a paucity of data on whether TGF-*β*2 and TGF-*β*3 contribute to the susceptibility and the severity of atherosclerosis in CKD patients.

The* TGF-β1* gene, located on the long arm of chromosome 19, contains six common single nucleotide polymorphisms (SNPs), namely, C-988A, G-800A, C-509T, T-869C, G-915C, and C-11929T [24, 25]. Previous studies have shown that* TGF-β1* gene polymorphisms predicted serum levels of TGF-*β*1 [26–28]. However, the role of* TGF-β1* gene polymorphisms in atherosclerotic cardiovascular disease in CKD patients remains controversial. While some studies have linked polymorphisms in the genes encoding TGF-*β*1 to increased risk of atherosclerosis [29–31], studies in other populations were negative [32–34]. It is against this background that we performed measurements of serum levels of TGF-*β* isoforms concurrently with serum levels of endotoxin and some inflammatory markers (lipoprotein binding protein, serum CD14, and monocyte chemoattractant protein-1) and examined anti-inflammatory and atheroprotective effects of TGF-*β* isoforms in South African CKD patients. In addition, we determined if any association exists between polymorphisms in the* TGF-β1* gene and atherosclerosis in South African CKD patients.

## 2. Materials and Methods

### 2.1. Study Population

The study was approved by the University of the Witwatersrand, Human Research Ethics Committee. A total of 160 participants, comprising 40 stage 3 CKD patients, 40 peritoneal dialysis (PD) patients, 40 haemodialysis (HD) patients, and 40 controls, were included in this study. Exclusion criteria included clinical signs of active or chronic infection, diabetes mellitus, seropositive status for hepatitis B, C and HIV, autoimmune disease, liver dysfunction, malignancy, heart failure, and use of anti-inflammatory or immunosuppressive therapy at least three months prior to enrolment. Using a structured interview form, information on age, race, gender, and tobacco use was documented. Patients were classified as smokers if they were current smokers, former smokers if they stopped smoking for at least six months prior to the study, and nonsmokers if they had never smoked.

### 2.2. Blood Pressure Measurement

Blood pressure for HD patients was recorded noninvasively in the arm without the A-V fistula with an Accoson mercury sphygmomanometer in the sitting position before a dialysis session commenced. Blood pressure was estimated by averaging all pre-dialysis and post-dialysis blood pressure recordings taken during the month before the study (3 measurements per week for a total of 12 measurements, that is, 3/week). Among PD and CKD patients, blood pressure was recorded at the time of the clinic visit. The blood pressure average of four clinic visits was taken as the patient's actual BP. In control patients, blood pressure was measured in the sitting position after resting for 5 minutes and an average of three readings recorded 5 minutes apart was used. Pulse pressure was calculated as systolic blood pressure (SBP) minus diastolic blood pressure (DBP). Mean arterial blood pressure (MABP) was calculated as diastolic blood pressure plus one-third pulse pressure.

### 2.3. Blood Sample Collection

Following an overnight fast, 10mls of blood was collected into anticoagulant-free tubes and kept on ice until the serum was separated within 30 minutes of collection and centrifuged at 3000 rpm for 10 minutes at room temperature. Serum was subsequently separated and stored in appropriate endotoxin-free Eppendorf tubes at -70°C until analysis. Serum creatinine, albumin, total cholesterol, high density lipoprotein (HDL), low density lipoprotein (LDL) and triglyceride (TG) levels were measured using ADVIA^R^ auto-analyzers (Siemens Healthcare Diagnostics Inc, USA).

### 2.4. Transforming Growth Factor-*β*1, *β*2, and *β*3 Concentrations

Serum TGF-*β*1, 2, and 3 levels were determined using BioPlex Pro^(TM)^ TGF-*β* Assay kits (Bio-RAD Laboratories, Inc., Hercules, CA, USA). Assays were carried out in accordance with the manufacturer's instructions. The sample dilution was 1:16. Fluorescence was measured on the Bio-Plex^(TM)^ 200 system (Bio-Rad) and concentrations were generated automatically with Bio-Plex manager software, version 5.0 (Bio-Rad Laboratories Inc).

### 2.5. Endotoxin Levels

Circulating endotoxin was measured using the Limulus amebocyte lysate QCL-1000 assay (Lonza, Walkersville, USA) according to manufacturer's instructions, using a previously described method [34]. Absorbance was measured using an ELx800 microplate reader (BioTek Instruments, Inc, VT, USA).

### 2.6. Lipopolysaccharide Binding Protein (LBP) Concentrations

Lipopolysaccharide binding protein levels were assayed using a commercial human LBP ELISA kit, Hycult HK315 (Hycult Biotechnology, Uden, Netherlands), in accordance with the manufacturer's instructions.

### 2.7. Inflammatory Marker Assays

Serum high sensitivity C-reactive protein (hs-CRP), serum CD14 (sCD14), and monocyte chemoattractant protein 1 (MCP-1) were analyzed using Luminex® Performance Assay multiplex kits (R&D Systems, Inc., Minneapolis, USA). Assays were carried out in accordance with the manufacturer's instructions. For hs-CRP measurements, the sample dilution was 1:1000 while samples for sCD14 measurements were diluted 1:50. Samples for MCP-1 were not diluted. Fluorescence was measured on the Bio-Plex™ 200 system (Bio-Rad) and concentrations were generated automatically with Bio-Plex manager software, version 5.0 (Bio-Rad Laboratories Inc).

### 2.8. Carotid Intima Media Thickness Measurement

Carotid intima media thickness was assessed using high resolution B-mode ultrasonography with the aid of L3-11 MHz linear array transducer (Philips Corporation USA) according to American Society of Echocardiography guidelines [35, 36]. Carotid intima media thickness was measured in plaque-free areas. Carotid plaque was defined as the echogenic structure protruding into the lumen with the distance between the media adventitia interface and the internal side of the lesion ≥ 1.2 mm. All measurements were performed by the same sonographer who was blinded to the clinical details and laboratory data of the participants.

### 2.9. DNA Extraction and TGF-*β*1 Genotyping

All procedures were carried out at room temperature (15-25°C). Genomic DNA was extracted from whole blood using a modified salting out method as previously described [37]. Genotyping was performed on the study groups and the controls using a cytokine genotyping tray kit (One Lambda Inc., Los Angeles, USA). The preoptimized primers were presented lyophilised in different wells of a 96-well 0.2ml thin-walled tube tray for polymerase chain reaction (PCR), to which DNA samples (100 ng), recombinant Taq polymerase (5U/*μ*L HotStarTaq DNA Polymerase, Qiagen, Hilden, Germany), and specially formulated dNTP-buffer mix (D-mix) were added as per the manufacturer's protocol. The PCR products were amplified on a thermocycler (MJ Mini Thermal cycler, Bio-Rad) using the One Lambda PCR program (1 cycle: 96°C for 120s; 10 cycles: 96°C for 10s, 63°C for 60s; 20 cycles: 96°C for 10s, 59°C for 50s, 72°C for 30s; hold at 4°C) according to manufacturer's instructions. All the PCR products were visualized on a 2% agarose gel stained with ethidium bromide, with the aid of an image analyzer (Gel Doc™ EZ Imager, Bio-Rad).* TGF-β1* SNPs were assessed based on the sizes of the amplified products with negative amplifications scored only if the internal control product was present.

### 2.10. Data Analysis

Data analyses were performed using the statistical package for social sciences (SPSS) 16 (SPSS, Inc., Chicago IL). Variables were presented as mean ± SD and median (interquartile range, IQR) for normally and nonnormally distributed continuous data, respectively, and percentages and frequencies for categorical data. Results were analyzed using a* t*-test with the Tukey post hoc test for normally distributed data and the chi-square test and Kruskal Wallis test for nonparametric data. Correlation between variables was assessed by the Spearman correlation coefficients. Genotype frequencies were determined by gene counting method and expressed as percentages. The frequencies were compared using Fisher's exact test. Further analysis was performed to assess the influence of various genotypes of* TGF-β1* on the serum levels of TGF-*β*1 and CIMT. A P-value <0.05 (two-tailed) was considered significant.

## 3. Results

### 3.1. Demographic and Clinical Data

Patients' demographics and clinical and laboratory data are shown in Table 1. This study consisted of 120 patients comprising PD, HD, and stage 3 CKD patients, with mean ages of 40.6±9.9, 40.6±10.1, and 42.1±10.6 years, respectively. In each group, male patients comprised 55% of the studied population. Of the patients, 106 (88.3%) were Black, 8 (6.7%) were White, 3 (2.5%) were Indian, and 3 (2.5%) were of mixed race. The controls were matched for age and gender. There were 22 (55%) male and 18 (45%) female controls. The mean age for the controls was 42.2±10.1 years. The aetiology of CKD was hypertension-attributed in 59/120 (49.2%), chronic glomerulonephritis in 36/120 (30%), polycystic kidney disease in 8/120 (6.7%), reflux nephropathy in 4/120 (3.3%), congenital abnormalities of the kidneys in 4/120 (3.3%) patients, obstructive uropathy in 3/120 (2.5%), and unknown in 7 (5%) patients. Hypertension as the primary cause of CKD was present in 52/59 (88.1%) of black patients and only in 7/52 (11.9%) in other race groups.

One hundred and two patients (85%) received various combinations of antihypertensive agents. Calcium channel blockers were taken by 81/120 (67.5%) patients, beta blockers by 40/120 (33.3%), angiotensin-II receptor blockers or angiotensin-converting enzyme inhibitors (ARB/ACEI) by 27/120 (22.5%), diuretics by 22/120 (18.3%), and alpha blockers by 16/120 13.3%) of the patients. Regarding patients that were treated with antihypertensive medications, 43/102 (42.2%) were on monotherapy, 39/102 (38.2%) on double, 15/102 (14.7%) on triple, and 3/102 (2.9%) on quadruple agents while 2/102 (1.96%) patients received 5 agents in various combination. In addition, patients received other medications for ESRD management including phosphate binders in 104/120 (86.7%), statins in 28/120 (23.3%), and aspirin in 9/120 (7.5%) of cases.

### 3.2. Transforming Growth Factor-Beta Isoform Levels in CKD Patients

The median concentrations of the three TGF-*β* isoforms are presented in Table 1. Of the three TGF-*β* isoforms, TGF-*β*1 had the highest levels. The lowest TGF-*β* isoform concentrations were present in HD patients compared to the PD, CKD patients, and controls. Female CKD patients had significantly higher levels of TGF-*β*1 as compared to male patients (TGF-*β*1: 49.6 (41.2-60.2) ng/ml versus 39.4 (31.1-49.3) ng/ml, p=0.001). No relationship was found between any of the TGF-*β* isoforms and age. Both TGF-*β*1 and TGF-*β*3 isoforms levels were significantly lower in HD patients compared to other study groups including the controls (p<0.05), while, in the subanalysis of TGF-*β*2 concentrations, there was no difference between HD and PD patients (p>0.05). In CKD patients (HD, PD, and stage 3 CKD), TGF-*β* isoforms levels were not associated with the aetiology of the CKD. Even though angiotensin-converting enzyme inhibitor was previously shown to lower serum TGF-*β*1 levels in patients with diabetic nephropathy [38, 39], this current study, however, did not show any significant differences in TGF-*β* isoforms levels between CKD patients who were treated with ARB/ACEI and those not treated with ARB/ACEI [TGF-*β*1: 46.7 (36.7 – 53.6) versus 44.1 (33.7 – 65.1) ng/ml, p=0.259; TGF-*β*2: 1.63 (1.44 – 1.85) versus 1.68 (1.50 – 1.86) ng/ml, p=0.453; and TGF-*β*3: 0.47 (0.43 – 0.57) versus 0.44 (0.38 – 0.50), p=0.120 ng/ml]. As shown in Table 1, CKD patients had significantly lower concentrations of TGF-*β*1 and TGF-*β*3 compared to the controls (p<0.001), while there was no difference in the concentration of TGF-*β*2 between CKD patients and controls (p=0.062).

### 3.3. Relationship Between TGF-*β*, Inflammatory Cytokines, Lipoprotein Particles, and Blood Pressure

Table 2 shows the relationship between TGF-*β* isoforms, renal function, inflammation, and CIMT. When TGF-*β*1 was correlated with mediators of the endotoxin signalling pathway, a modest relationship was demonstrated between TGF-*β*3 and LBP (r=−0.403, p<0.001), and serum CD14 (r=−0.318, p<0.001). Transforming growth factor-*β*1 showed a weak relationship with MCP-1 (r=−0.212, p=0.020). Transforming growth factor-*β*3 demonstrated a weak negative correlation with hs-CRP (r=−0.320, p<0.001) while TGF-*β*1 showed an inverse relationship with MABP (r=−0.248, p=0.006). TGF-*β*3 showed a weak positive correlation with albumin (r=0.256, p=0.005). Furthermore, TGF-*β*3 had a positive correlation with total cholesterol, LDL, HDL, and TG. Multiple linear regression analysis showed that CIMT and MABP were independent predictors of TGF-*β*1 levels (r^2^=0.41, p<0.001) (Table 3) and CIMT and MCP-1 were independent determinants of serum TGF-*β*2 (r^2^=0.39; p<0.001) while CIMT was the only predictor of serum TGF-*β*3 levels (r^2^=0.33; p<0.001).

### 3.4. Atherosclerosis and Transforming Growth Factor-*β* Isoforms

**Figure 1:**
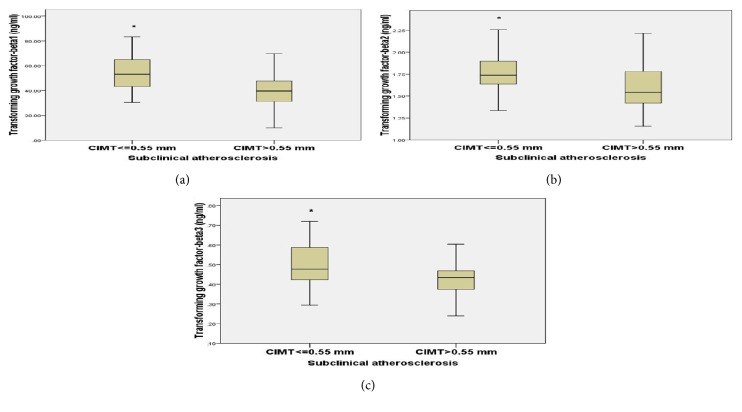
**Comparison of serum transforming growth factor-** **β** **1 (a),** **β** **2 (b), and** **β** **3 (c) between patients with subclinical atherosclerosis and those without atherosclerosis.** The boxes indicate median, 25th, and 75th percentile; whiskers represent data range; whisker caps indicate 5th and 95th percentile. Transforming growth factor-*β*1, *β*2, and *β*3 levels were analyzed with Bio-Plex Pro™ TGF-*β* Assays kit. Carotid intima media thickness was measured using B-mode ultrasound. Serum transforming growth factor-*β*1, *β*2, and *β*3 levels were compared between CKD patients and controls, *∗* P < 0.001 compared to controls.

**Figure 2:**
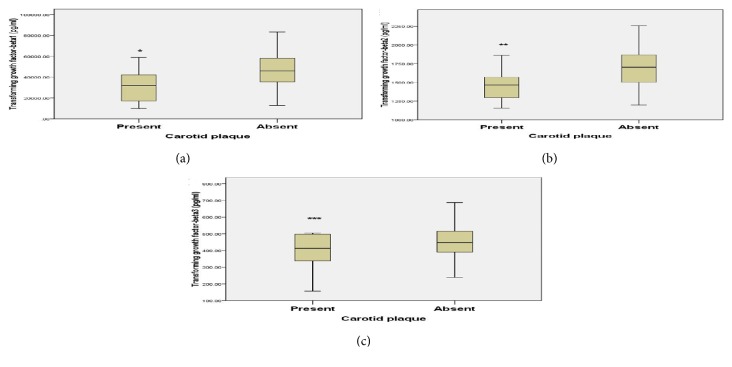
**Comparison of serum transforming growth factor-** **β** **1 (a),** **β** **2 (b), and** **β** **3 (c) between patients with carotid plaque and those without plaques.** The boxes indicate median, 25th, and 75th percentile; whiskers represent data range; whisker caps indicate 5th and 95th percentile. Transforming growth factor-*β*1, *β*2, and *β*3 levels were analyzed with Bio-Plex Pro™ TGF-*β* Assays kit. Carotid plaque was defined as the echogenic structure protruding into the lumen with the distance between the media adventitia interface and the internal side of the lesion ≥ 1.2 mm. Serum transforming growth factor-*β*1, *β*2, and *β*3 levels were compared between patients with carotid plaques and those without plaque, *∗*P = 0.016, *∗∗* P = 0.013, and *∗∗∗*P = 0.330 compared to those patients without plaque.

Sixty-seven CKD patients (55.8%) had subclinical atherosclerosis (CIMT of > 0.55 mm). Carotid plaques were present in 5% of PD, 12.5% of HD, and 2.5% of nondialytic CKD patients, but not in any of the controls (Table 1). Transforming growth factor-*β* isoforms concentrations were significantly lower in the patients with subclinical atherosclerosis compared to patients without atherosclerosis [TGF-*β*1: 39.1 (30.6 – 47.5) versus 53.9 (44.1 – 65.1) ng/ml, p<0.001; TGF-*β*2: 1.51 (1.42 – 1.73) versus 1.83 (1.64 – 1.96) ng/ml, p<0.001; and TGF-*β*3: 0.43 (0.37 – 0.46) versus 0.50 (0.42 – 0.62), p<0.001 ng/ml] (Figure 1). Furthermore, TGF-*β*1 and TGF-*β*2 levels were significantly lower in patients with carotid plaque compared to those without carotid plaque [TGF-*β*1: 31.9 (17.2 – 42.2) versus 45.9 (35.4 – 58.1) ng/ml, p=0.016; and TGF-*β*2: 1.46 (1.30 – 1.57) versus 1.70 (1.50 – 1.87) ng/ml, p=0.013] (Figure 2). However, there was no difference in the levels of TGF-*β*3 between patients with carotid plaque and those without plaque [0.41 (0.34 – 0.50) versus 0.44 (0.39 – 0.52) ng/ml, p=0.330] (Figure 2). Overall, TGF-*β* isoforms had inverse relationships with CIMT (Table 2). Age, smoking, MABP, HDL, LDL, TG, hs-CRP, serum creatinine (marker of kidney function), TGF-*β*1, TGF-*β*2, and TGF-*β*3 levels were entered into the multiple logistic regression analysis as covariates to determine their contribution to the risk of atherosclerosis. Age, TGF-*β*2, and TGF-*β*3 were the only independent predictors of subclinical atherosclerosis in CKD patients in the regression model [age: Odds ratio (OR), 1.054; 95% confidence interval (CI): 1.003 – 1.109, p=0.039; TGF-*β*2: OR, 0.996; 95% CI: 0.994–0.999, p=0.018; TGF-*β*3: OR, 0.992; 95% CI: 0.985–0.999, p=0.029) (Table 4).

### 3.5. Circulating Endotoxaemia and CIMT in CKD Patients

Carotid intima media thickness was significantly greater in CKD patients (median, 0.60 mm; IQR, 0.47-0.61 mm) compared to controls (median, 0.40 mm; IQR, 0.42-0.52 mm), p<0.001. Patients with elevated circulating endotoxaemia (> 0.5 EU/ml) had significantly higher CIMT compared to patients with lower endotoxin levels (≤ 0.5 EU/ml) (p<0.001). Carotid intima media thickness correlated with endotoxin (r=0.313, p=0.001) and LBP (r=0.311, p=0.001). On univariate analysis, atherosclerosis was associated with endotoxin levels (OR, 4.16; 95% CI: 1.04 – 16.6), p=0.044), with excess risk confined to the group with high endotoxin levels.

### 3.6. TGF-*β*1 Polymorphisms, TGF-*β*1 Levels, and Atherosclerosis

The distribution of the* TGF-β1* SNPs [T-869C (rs1800470) and G-915C (rs1800471)] and their genotyping frequencies in the CKD patients and the controls are shown in Table 5. The* TGF-β1* genotypes did not differ between controls and the CKD patients (p>0.05). Further analysis was done to determine whether the presence of* TGF-β1* genotypes influence the levels of TGF-*β*1 in the sera of the study participants. Although serum levels TGF-*β*1 were higher among the high producers compared to the intermediate producers and the low producers, they were not statistically significant (Table 6). In the CKD group, no association was found between the* TGF-β1* genotypes and subclinical atherosclerosis (Table 7).

## 4. Discussion

This study has demonstrated that serum levels of TGF-*β*1, TGF-*β*2, and TGF-*β*3 are significantly reduced in CKD patients compared to the control group, especially in patients with subclinical atherosclerosis and carotid plaque. This is in agreement with previous studies in non-CKD patients, stage 3 CKD patients, and dialysis patients [10, 28, 40]. It has also been demonstrated that there is reduced expression of TGF-*β*1 by peripheral leucocytes in patients who had acute myocardial infarction [41]. Furthermore, our finding is also in support of a previous study that showed that TGF-*β*1 expression inversely correlated with ankle-brachial index (another surrogate marker of atherosclerosis) in patients with peripheral arterial disease [16]. In agreement with our observations, Janda and colleagues identified age and TGF-*β*1 as independent predictors of common carotid artery intima media thickness (CCA-IMT) among end-stage renal disease patients treated with peritoneal dialysis; however, they observed a positive correlation between TGF-*β*1 and CCA-IMT [42].

The inverse relationship between TGF-*β* isoforms and accelerated atherosclerosis in the CKD patients may be related to the antiproliferative and cardioprotective properties of these immunomodulatory cytokines. Transforming growth factor-*β*1, the most extensively studied of the three closely related isoforms of TGF-*β*, counteracts vascular inflammation by inhibiting the synthesis of tumour necrosis factor-*α* [14]. Furthermore, by downregulating the proinflammatory effects of IL-1*β* and interferon-*γ*, it leads to reduction of inflammatory cytokine-induced VCAM-1, chemotaxis, leucocyte adhesion to vascular endothelial lining, and decreased macrophage activity [19, 21]. Thus, TGF-*β*1 is important in the maintenance of normal vascular integrity.

Transforming growth factor-*β*1 has been shown previously by Arciniegas and colleagues to induce the differentiation of aortic endothelial cells into contractile, synthetic, and luminal smooth muscle cells in TGF-*β*1-treated cultures [43]. The authors further demonstrated that TGF-*β*1 inhibited cell proliferation and induced morphological changes, resulting in decreased expression of factor VIII-related antigen and increased expression of *α*-smooth muscle actin (contractile protein) in smooth muscle cells which, in turn, play a vital role in the maintenance of healthy blood vessels [43]. Taken together, these* in vitro* functions of TGF-*β*1 are consistent with the hypothesis that TGF-*β*1 may play a role in the process of atherogenesis.

There is no consensus about the role of TGF-*β*1 in the process of atherosclerosis and restenosis. Some studies have reported an association between elevated TGF-*β*1 levels and vascular restenosis lesions [30, 44]. In contrast, other authors have showed that decreased expression of TGF-*β*1 contributes to progression of atherosclerosis [10, 22, 23]. Nevertheless, the absence of the antiproliferative effects of TGF-*β*1 in the blood vessels leads to increased chemotaxis, deposition of extracellular matrix, proliferation of vascular smooth muscle cells, and decreased apoptosis, thereby facilitating progression of atherosclerosis [45, 46]. Moreover, the negative associations between TGF-*β* isoforms and inflammatory mediators (LBP, sCD14, MCP-1) observed in the current study further highlight the anti-inflammatory effect of TGF-*β*1.

In this study, serum levels of TGF isoforms predicted reduced risk for subclinical atherosclerosis in patients with CKD. These findings are compatible with the hypothesis that TGF-*β*, an anti-inflammatory cytokine, is implicated in the pathogenesis of atherosclerosis [40, 47]. However, our study rules out the possibility that biologic variations in* TGF-β1* gene affect serum levels of TGF-*β*1 and the development of atherosclerosis. This result suggests that, given the complexity and the variety of the TGF-*β* superfamily of ligands, receptors, and binding proteins,* TGF-β1* gene polymorphisms alone may not sufficiently explain the reduced susceptibility and severity of atherosclerosis observed among studied CKD patients. Therefore, future studies targeted at exploring potential defects in the activation and signalling pathway of TGF-*β* might well hold the key to understanding the mechanisms leading to low serum TGF-*β* isoform levels in CKD patients with atherosclerotic CVD.

Hypertension, an established risk factor for myocardial infarction, showed weak but significant association with TGF-*β*1 levels. This observation was supported by the report of an inverse relationship between* TGF-β1* polymorphisms/hypomorphs and hypertension in previous human and animal studies [48, 49]. Likewise, gender to some extent affected TGF-*β*1 concentrations in this study. This finding is in support of a previous study in non-CKD patients that reported an association between TGF-*β*1 levels and gender [50]. The authors postulated that serum TGF-*β*1 levels in women may be under the control of antioestrogen hormones, ultimately resulting in the secretion of TGF-*β*1 by fetal human fibroblasts.

The finding that TGF-*β*1 levels demonstrated a modest significant correlation with hs-CRP and albumin (a marker of malnutrition) was consistent with the report of Stefoni et al. [40]. Furthermore, previous studies had suggested a link between malnutrition, inflammation, and cardiovascular disease morbidity and mortality in end-stage renal disease patients [51–54]. Therefore, the association between serum TGF-*β*1 levels and hs-CRP may suggest the degree of vascular inflammation, while correlation with low serum albumin may suggest a state of malnutrition which is very common in CKD patients [35].

The low levels of TGF-*β* in haemodialysis patients observed in this study may be due to subclinical endothelial damage or a result of heparin-mediated activation of TGF-*β* signalling pathways leading to exhaustion of TGF-*β* from the repeated binding of TGF-*β* to various TGF-*β* receptors [51]. However, heparin-mediated activation of TGF-*β* pathways does not explain the low levels of TGF-*β* in peritoneal dialysis and stage 3 CKD patients, since these groups of patients are not exposed to heparin. Moreover, multiple linear regression analysis showed that subclinical atherosclerosis is an independent determinant of TGF-*β* levels in all CKD patients. It is therefore plausible that subclinical endothelial damage leading to progression of atherosclerosis may provide an explanation for the lower levels of TGF-*β* isoforms in the CKD patients compared to the controls. Nonetheless, additional studies are needed to explore the complex biology of TGF-*β* signalling pathways in CKD patients.

There are some important limitations of our study. Firstly, the sample size is relatively small. This may have limited the statistical power of the study to detect any association between TGF-*β*1 polymorphisms and serum TGF-*β*1 levels as well as subclinical atherosclerosis. A larger study in a more diverse CKD population in sub-Saharan Africa is needed to determine if our findings are generalizable. A second important limitation is that the study design was essentially a cross-sectional one and the measurements were only carried out at one point. Therefore, our results can only be regarded as preliminary. A prospective epidemiological study is needed to determine the potential protective role of TGF-*β* on the risk of incident atherosclerosis in the African populations.

In conclusion, we demonstrated that serum levels of TGF-*β* isoforms were significantly lower in patients with subclinical atherosclerosis and predicted reduced risk for subclinical atherosclerosis in South African patients with CKD. Given the cross-sectional design of this study, the cause and effect relationship between serum TGF-*β* isoform levels and atherosclerosis remains to be established. In this context, low serum TGF-*β* isoforms levels can only be considered an important, but not a sufficient risk factor for inflammation-related atherosclerosis in CKD patients. Future prospective longitudinal controlled studies will be needed to evaluate the role of TGF-*β*1 on the risk of incident atherosclerotic CVD among indigenous African CKD populations.

## Figures and Tables

**Table 1:** Demographics and clinical and laboratory data of the study population.

Parameter	All patients (N=120)	PD (N=40)	HD (N=40)	CKD stage 3 (N=40)	Control (N=40)	*∗*P value
Age (years; mean ± SD)	41.1 ± 10.2	40.6 ± 9.9	40.6 ± 10.1	42.1 ± 10.6	42.2 ± 10.1	0.494^a^
Sex (Male/Female)	60/40	22/18	22/18	22/18	22/18	0.784^b^
Race (Black/Non-Black)	106/14	37/3	37/3	35/5	31/9	0.091^b^
Smoking (Yes/No)	17/103	9/31	5/35	3/37	2/38	0.121^b^
MABP (mmHg)	134.8 (118.8-150.0)	143.3 (130.0-163.8)	144.7 (135.2-157.3)	136.2 (119.5-148.6)	117.0 (107.8-130.6)	<0.001^c^
Serum creatinine (mmol/L)	262.5 (92.3-766.0)	1175.5 (877.0-1363.0)	513.0 (401.5-727.0)	124.0 (105.5-166.8)	71.5 (60.5-86.0)	<0.001^c^
eGFR (ml/min/1.73m^2^)	-	NA	NA	47.5 (44.0-50.0)	96.5 (83-119)	-
Serum albumin (g/L)	40.0 (36.0-43.0)	35.5 (33.0-40.0)	38.5 (35.0-41.0)	41.5 (38.3-44.0)	43.0 (40.3-44)	<0.001^c^
Total cholesterol (mmol/L)	4.20 (3.43 – 5.18)	5.20 (4.20 – 5.90)	3.40 (3.00 – 3.70)	4.40 (4.00 – 5.10)	4.00 (3.33 – 4.88)	0.519^c^
HDL (mmol/L)	1.20 (0.90 – 1.40)	1.2 (0.90 – 1.40)	1.00 (0.90 – 1.20)	1.30 (1.10 – 1.40)	1.25 (1.03 – 1.40)	0.421^c^
LDL (mmol/L)	2.30 (1.80 – 3.00)	3.00 (2.40 – 4.10)	1.80 (1.40 – 2.20)	2.50 (1.90 – 3.00)	2.20 (1.60 – 2.98)	0.456^c^
TG (mmol/L)	1.20 (0.80 – 1.70)	1.30 (1.00 – 2.00)	0.80 (0.60 – 1.20)	1.30 (1.00 – 1.90)	0.95 (0.63 – 1.60)	0.172^c^
TGF-*β*1 (ng/ml)	45.0 (33.5-56.9)	45.1 (33.8-52.3)	36.0 (25.2-44.4)	59.0 (46.1-66.9)	66.3 (57.7-75.4)	<0.001^c^
TGF-*β*2 (ng/ml)	1.7 (1.5-1.9)	1.6 (1.4-1.9)	1.6 (1.5-1.8)	1.7 (1.5-1.9)	1.8 (1.6-1.9)	0.062^c^
TGF-*β*3 (ng/ml)	0.45 (0.39-0.50)	0.44 (0.38-0.50)	0.40 (0.36-0.45)	0.48 (0.44-0.60)	0.52 (0.48-0.65)	<0.001^c^
MCP-1 (pg/ml)	9.0 (4.8-17.0)	12.3 (7.2-19.3)	7.2 (4.5-10.9)	6.8 (4.6-12.2)	3.6 (2.2-7.3)	<0.001^c^
sCD14 (*μ*g/ml)	1.8 (1.3-2.2)	2.0 (1.5-2.6)	1.8 (1.4-2.1)	1.5 (1.0-1.9)	1.2 (1.0-1.4)	<0.001^c^
Hs-CRP (mg/dL)	0.7 (0.2-1.4)	1.1 (0.5-1.9)	1.0 (0.4-1.6)	0.7 (0.2-1.4)	0.2 (0.1-0.7)	<0.001^c^
Endotoxin (EU/ml)	0.46 (0.30-0.67)	0.56 (0.44-0.75)	0.51 (0.28-0.78)	0.52 (0.31-0.70)	0.33 (0.26-0.41)	<0.001^c^
LBP (ng/ml)	1.2 (0.9-1.6 )×10^5^	1.4 (1.2-1.7)×10^5^	1.4 (1.1-1.7) ×10^5^	1.2 (0.9-1.4) ×10^5^	0.9 (0.8-1.1) ×10^5^	<0.001^c^
CIMT (mm)	0.6 (0.47-0.61)	0.6 (0.54-0.71)	0.5 (0.49-0.61)	0.5 (0.47-0.61)	0.4 (0.42-0.52)	<0.001^c^
Plaques (Present; %)	8 (6.7%)	2 (5%)	5 (12.5%)	1 (2.5%)	0 (0%)	<0.001^b^

PD, peritoneal dialysis; HD, haemodialysis; CKD, chronic kidney disease; MABP, mean arterial blood pressure; HDL, high density lipoprotein; LDL, low density lipoprotein; TG, triglycerides; MCP-1, monocyte chemoattractant protein-1; sCD14, serum CD14; TGF, transforming growth factor; Hs-CRP, high sensitivity C-reactive protein; LBP, lipopolysaccharide binding protein; CIMT, carotid intima media thickness. Continuous data were expressed as mean ± SD or median (IQR) and categorical data as percentages. *∗* P-values compare all CKD patients (n=120) to controls (n=40).

^a^p-value calculated using Student's *t*-test.

^b^p-values calculated using Chi-square test.

^c^p-values calculated using Mann–Whitney test.

**Table 2:** Correlation between transforming growth factor *β* isoforms, renal function, lipoprotein particles, inflammation, and markers of atherosclerosis.

Variables	TGF-*β*1		TGF-*β*2		TGF-*β*3	
	r value	p value	r value	p value	r value	p value
MABP	− 0.248	0.006	− 0.028	0.763	− 0.136	0.137
Serum creatinine	− 0.348	< 0.001	− 0.237	0.009	− 0.316	< 0.001
Serum albumin	0.247	0.007	0.192	0.036	0.257	0.005
Total cholesterol	0.322	< 0.001	0.226	0.013	0.356	< 0.001
HDL	0.258	0.004	0.262	0.004	0.229	0.012
LDL	0.248	0.006	0.155	0.091	0.263	0.004
TG	0.221	0.016	0.142	0.121	0.269	0.003
Hs-CRP	− 0.183	0.046	− 0.153	0.096	− 0.320	0.001
MCP-1	− 0.212	0.020	− 0.069	0.457	− 0.184	0.045
sCD14	− 0.347	< 0.001	− 0.313	0.001	− 0.318	< 0.001
Endotoxins	− 0.196	0.032	− 0.207	0.023	− 0.139	0.130
LBP	− 0.281	0.002	− 0.402	< 0.001	− 0.403	< 0.001
CIMT	− 0.614	< 0.001	− 0.547	< 0.001	− 0.430	< 0.001

MABP; mean arterial blood pressure; HDL, high density lipoprotein; LDL, low density lipoprotein, TG, triglycerides; Hs-CRP, high sensitivity C-reactive protein; MCP-1, monocyte chemoattractant protein-1; sCD14, serum CD14, LBP, lipopolysaccharide binding protein; CIMT, carotid intima media thickness; TGF, transforming growth factor. Correlation was assessed by Spearman's correlation coefficient.

**Table 3:** Multiple linear regression analysis of determinants of serum TGF-*β*1 levels.

Variables	Unstandardized coefficients (*β*)	Standardized coefficients (Beta)	95% Confidence interval	P value
CIMT	− 81439.078	− 0.593	− 107604.8 – −55273.3	< 0.001
Creatinine	− 3.111	− 0.095	− 8.484 – 2.262	0.254
sCD14	0.000	– 0.006	– 0.005 – 0.005	0.965
LBP	– 0.026	– 0.096	– 0.082 – 0.031	0.374
MABP	– 122.815	– 0.174	– 226.892 – – 18.739	0.021
Albumin	304.912	0.095	– 227.427 – – 837.251	0.259
MCP-1	258.890	0.181	– 13.286 – 531.066	0.062
Hs-CRP	– 230.671	– 0.011	– 3758.684 – 3297.342	0.897

CIMT, carotid intima media thickness; MABP; mean arterial blood pressure; sCD14, serum CD14; LBP, lipopolysaccharide binding protein; MCP-1, monocyte chemoattractant protein-1; Hs-CRP, high sensitivity C-reactive protein.

**Table 4:** Multiple logistic regression analysis of risk factors for atherosclerosis in CKD patients.

Variables	Β	Standard error of *β*	Odds ratio	95% Confidence interval	P value
Age	0.053	0.026	1.054	1.003 – 1.109	0.039
Smoking	0.961	0.775	2.615	0.573 – 11.939	0.215
MABP	− 0.005	0.011	0.995	0.974 – 1.017	0.678
HDL	− 0.436	0.564	0.647	0.214 –1.953	0.440
LDL	0.392	0.269	1.480	0.874 – 2.507	0.144
TG	0.579	0.371	1.784	0.862 – 3.690	0.119
Hs-CRP	– 0.505	0.356	0.604	0.300 – 1.213	0.156
Serum Creatinine	0.000	0.001	1.000	0.998 – 1.001	0.468
TGF-*β*1	0.000	0.000	1.000	1.000 – 1.000	0.046
TGF-*β*2	– 0.004	0.001	0.996	0.994 – 0.999	0.018
TGF-*β*3	– 0.008	0.004	0.992	0.985 – 0.999	0.029

HDL, high density lipoprotein; LDL, low density lipoprotein, TG, triglycerides; Hs-CRP, high sensitivity C-reactive protein; TGF, transforming growth factor.

**Table 5:** *TGF-β1* T-869C and G-915C SNPs distribution and frequency in study participants.

SNPs /Producer	CKD patients (n=79)	Controls (n=32)	P-value
T/T G/G (high)	16 (20.3%)	7 (21.9%)	1.00
T/C G/G (high)	41 (51.9%)	17 (53.1%)	1.00
T/C G/C (intermediate)	6 (7.6%)	5 (15.6%)	0.29
C/C G/G (intermediate)	9 (11.4%)	1 (3.1%)	0.28
T/T G/C (intermediate)	1 (1.3%)	0 (0%)	1.00
C/C G/C (low)	5 (6.3%)	2 (6.3%)	1.00
T/C C/C (low)	1 (1.3%)	0 (0%)	1.00

TGF-*β*1, transforming growth factor *β*1; T-869C, rs1800470; G-915C, rs1800471. P-values were calculated using Chi-square and Fisher's exact test where applicable. From the analysis of the SNPs, there was no difference between CKD patients and the controls (p>0.05).

**Table 6:** Producer status and transforming growth factor-*β*1 in genotyped study participants (n=111).

**Producer status**	**High**	**Intermediate**	**Low**	**∗** **P-value**
Frequency	81 (73.0%)	22 (19.8%)	8 (7.2%)	0.649
TGF-*β*1 levels (ng/ml) (Median; IQR)	52.9 (41.8 – 64.6)	45.8 (35.8 – 68.0)	46.8 (42.8 – 82.5)	

TGF-*β*1: transforming growth factor *β*1. *∗*P-value was calculated using Kruskal-Wallis test and compared TGF-*β*1 levels across the three comparison groups. Post hoc analysis did not showed any significant difference among the three comparison groups (p>0.05).

**Table 7:** Relationship between *TGF-β1* gene polymorphisms (T-869C and G-915C) and atherogenesis in CKD patients.

***TGF-*** **β** ***1* SNPs /Producer**	**Odds Ratio**	**95**%** Confidence Interval**	**P-value**	**Risk of Atherogenesis**
T/T G/G (high)	1.26	0.53-3.01	0.406	No association
T/C G/G (high)	0.98	0.64-1.51	0.560	No association
T/C G/C (intermediate)	6.29	0.77-51.4	0.057	No association
C/C G/G (intermediate)	0.36	0.08-1.62	0.144	No association
T/T G/C (intermediate)	1.02	0.98-1.07	0.562	No association
C/C G/C (low)	0.31	0.04-2.69	0.259	No association
T/C C/C (low)	0.97	0.91-1.03	0.440	No association

TGF*β*1: transforming growth factor *β*1. Odds ratios, 95% confidence interval, and p-value were derived from analyses of the strength of association between *TGF-β1* gene polymorphisms and subclinical atherosclerosis in CKD patients with atherosclerosis compared to those without atherosclerosis (reference group).
